# Association of Polycystic Ovarian Syndrome Features and Metabolic Syndrome Among Reproductive-Aged Women in the United States

**DOI:** 10.1089/whr.2024.0143

**Published:** 2025-04-14

**Authors:** Deepali K. Ernest, Asha Collier, Aparajita Chandrasekhar, Luyu Xie, Shaghayegh Darraji, Jenil Patel, Jaime P. Almandoz, Sarah E. Messiah

**Affiliations:** ^1^ Department of Epidemiology, School of Public Health, University of Texas Health Science Center at Houston (UTHealth), Houston, Texas, USA.; ^2^ School of Human Ecology, University of Texas at Austin, Austin, Texas, USA.; ^3^ Peter O’Donnell Jr. School of Public Health, University of Texas Southwestern Medical Center, Dallas, Texas, USA.; ^4^ School of Public Health, UTHealth at Houston, San Antonio, Texas, USA.; ^5^ School of Public Health, UTHealth at Houston, Dallas, Texas, USA.; ^6^ Department of Internal Medicine, University of Texas Southwestern Medical Center, Dallas, Texas, USA.; ^7^ Peter O’Donnell Jr School of Public Health, University of Texas Southwestern Medical Center, Dallas, Texas, USA.; ^8^ Department of Pediatrics, University of Texas Southwestern Medical Center, Dallas, Texas, USA.; ^9^ Children’s Health System of Texas, Dallas, Texas, USA.

**Keywords:** metabolic syndrome, polycystic ovarian syndrome, race/ethnicity, reproductive health

## Abstract

**Background:**

Polycystic ovarian syndrome (PCOS) is associated with the metabolic health of racially and ethnically diverse women globally, but limited research exists on the association of PCOS and metabolic syndrome (MetS) among women in the United States.

**Objective:**

To examine the association of PCOS features and MetS in a racially/ethnically diverse population of reproductive-aged women in the United States.

**Methods:**

Cross-sectional data from 2,172 women (12–49 years) from the 2011–2016 National Health and Nutrition Examination Survey were analyzed. Univariate logistic regression models determined unadjusted associations of MetS and its components (elevated central obesity, glucose, blood pressure and triglyceride, and low high-density lipoprotein cholesterol) with PCOS features (log-transformed total testosterone (LTT), sex-hormone binding globulin (LSHBG), amenorrhea, and oral contraceptive pills (OCP) use). Multivariable logistic models examined age-adjusted associations stratified by race and ethnicity.

**Results:**

The analytical sample (mean age = 30.3 years, 59% non-Hispanic White, 12.4% non-Hispanic Black, 18.7% Hispanic/Latina, 6.2% non-Hispanic Asian, 3.7% Other/multi-race) had a MetS prevalence of 14.5%. Overall, MetS was associated with age, body mass index, race/ethnicity, LTT and LSHBG concentrations, amenorrhea, and OCP use (*p* < 0.01 for all), and many of the PCOS features were protective against individual MetS components. Most race/ethnicities showed significantly lower odds of MetS with an increase in LSHBG, with varying impacts on individual MetS features.

**Conclusions:**

Findings suggest significant associations between PCOS features and MetS among a racially and ethnically diverse population of reproductive-aged women in the United States. More robust and longitudinal studies are needed to further understand the underlying mechanism linking PCOS and MetS.

## Introduction

Polycystic ovary syndrome (PCOS) is the most common endocrine disorder affecting reproductive-age women.^1^ Research suggests that 7%–13% of women globally are impacted by PCOS and that 70% of these cases are undiagnosed.^1,2^ The prevalence of PCOS within the United States is 7%–10% with 5 million American women living with PCOS.^3^ This condition disproportionately affects women of various races and ethnic backgrounds, with Hispanic (6.8%) and Native American (6.9%) women experiencing a disproportionate burden of PCOS and more severe cardiometabolic health complications compared to non-Hispanic white (NHW) women.^4–6^ Currently, a diagnosis of PCOS can be made using the Rotterdam Criteria,^7–12^ which require the presence of at least two of the following characteristics for a positive diagnosis: irregular menstrual cycles, clinical or biochemical evidence of excess androgen levels, or multiple cysts on the ovaries.

Irregular menstrual or anovulatory cycles, characterized by unpredictable timing, duration, or flow of menstruation, are key features of PCOS.^1^ This irregularity stems from hormonal imbalances, particularly elevated levels of androgens, such as testosterone, and insulin, which disrupt the normal ovulatory process.^1,8^ Without regular ovulation, menstrual cycles become irregular or absent, contributing to fertility challenges and other associated symptoms of PCOS.^1^ While there are several treatment options, oral contraceptive pills (OCP) are commonly prescribed to regulate menstrual cycles and manage androgen levels, helping to restore a more predictable menstrual pattern and reduce PCOS symptoms such as hirsutism and acne.^9^ Hyperandrogenism is also a defining feature of PCOS,^8^ which can manifest as acne, hirsutism, male-pattern baldness, and menstrual irregularity.^8^ Elevated androgens can result from disruptions in ovarian function, such as increased production of androgens by the ovaries or reduced clearance of androgens from circulation.^8^ Multiple cysts or follicles that fail to mature and release an egg during ovulation are commonly observed on the ovaries of individuals with PCOS.^1^ While the presence of ovarian cysts is a diagnostic criterion for PCOS, it is important to note that not all individuals with PCOS exhibit this feature.^8^ These hallmark features underscore the heterogeneous nature of PCOS, emphasizing the importance of a comprehensive approach to accurately diagnose and treat it.

While primarily known for its impact on reproductive health, PCOS represents a complex and multifaceted condition with far-reaching implications for women’s overall well-being. Beyond its effects on fertility and menstrual regularity, PCOS is increasingly understood to exert a significant influence on physical and mental health, while recently being recognized as a risk factor for cardiovascular mortality.^13–15^ Furthermore, its correlation with prevalent chronic diseases, such as obesity, type 2 diabetes, and cardiovascular disease, underscores the importance of timely detection and management of PCOS.^15,16^ However, disparities in healthcare access and cultural sensitivities can contribute to delayed diagnosis and suboptimal treatment, particularly among minority women, thereby increasing their susceptibility to long-term health complications associated with PCOS.^4–6^ Previous research in adults has shown an association between PCOS and cardiometabolic health, including metabolic syndrome (MetS), a cluster of three or more of the following: central obesity, fasting glucose, triglyceride, blood pressure, and low high-density lipoprotein (HDL) cholesterol, which in turn, is a significant risk factor for type 2 diabetes and cardiovascular disease.^16–19^ Indeed, the etiology of PCOS-associated cardiometabolic disturbances remains a subject of extensive research, with intricate hormonal and metabolic pathways involved. Reproductive hormonal imbalances, such as elevated luteinizing hormone (LH) relative to follicle-stimulating hormone, are distinguishing features of PCOS. At the same time, hyperandrogenism and insulin resistance are central contributors to the development of both PCOS and MetS.^19,20^ While previous research has shown that PCOS is an independent risk factor for MetS in adult females,^19,21^ the association between PCOS and MetS in reproductive-age adolescents and adults from racially and ethnically diverse populations is not as well understood. From a developmental science perspective, women’s reproductive years are critical for cardiometabolic and physiological development.^22^ Thus, early identification of risk factors for adverse reproductive and metabolic health outcomes is essential for the prevention and progression of such illnesses into later adulthood.

Therefore, our study aimed to examine the association between features of PCOS and MetS, as well as its individual components, in a racially and ethnically diverse population of reproductive-aged women (ages 12–49) in the United States.

## Methods

This cross-sectional study followed the strengthening the reporting of observational studies in epidemiology reporting guidelines.^23^ The University of Texas Health Science Center at Houston Committee for the Protection of Human Subjects ruled this study to be exempt from review because of the use of publicly available, de-identified data for analysis.

### Data source

The National Health and Nutrition Examination Survey (NHANES) 2011–2012, 2013–2014, and 2015–2016 data cycles were used for this analysis. NHANES is a cross-sectional survey conducted in a 2-year cycle by the National Center for Health Statistics to monitor the health and nutrition status of the US population.^24^ For each survey cycle, a complex 3-step weighting method was created by NHANES to represent the US noninstitutionalized, civilian population. The purpose of weighting the NHANES sample data is to permit the analysis of estimates that would have been obtained if the entire sampling frame had been surveyed. Weighting takes into account several features of the surveys: the specific probabilities of selection for the individual domains that were oversampled, as well as nonresponse and differences between the sample and the total population.^25^ The US National Center for Health Statistics Ethics Review Board approved the study (Protocol #2021-05).^26^

### Participant recruitment and data collection

NHANES participants were recruited through a multistage, representative sampling approach that involved the random selection of primary and secondary sampling units, followed by the recruitment of households and individuals to participate in comprehensive health examinations and interviews. NHANES face-to-face household interviews were conducted by trained interviewers using the computer-assisted personal interview system to gather information on demographics, medical conditions, and prescription medication use. Physical examinations, including anthropometric measures, and the collection of all biospecimens were conducted in the Mobile Examination Center by trained health workers. NHANES collected blood specimens of participants after fasting for at least eight hours. Triglyceride, fasting glucose, and HDL cholesterol were measured using laboratory methods detailed in the NHANES website.^25^ All participants 18 years or older completed written consent, while parental permission and child assent were obtained for participants younger than 18 years.

### Participant eligibility criteria

Female participants between the ages of 12 and 49 years who provided informed consent were not pregnant at the time of the exam, did not have diabetes, and had complete MetS individual component data available were included in the study. Participants with incomplete data were considered missing observations and excluded from the analysis. Based on these criteria, our final unweighted sample consisted of a total of 2,172 females.

### Assessment of metabolic syndrome (MetS)

MetS was diagnosed primarily based on the International Diabetes Foundation criteria.^27–29^ Briefly, MetS was defined as having central obesity (waist circumference ≥102 centimeters for males and ≥88 centimeters for females) and the presence of >2 of the following risk factors: (1) elevated systolic and diastolic blood pressure (SBP ≥130 mmHg and/or DBP ≥85 mmHg), (2) elevated fasting glucose (≥5.6 mmol/L), (3) Low HDL cholesterol (<40 mg/dL for males and females 12–15 years and males ≥16 years, <50 mg/dL for females ≥16 years), (4) elevated triglyceride (≥1.7 mmol/L). Each MetS feature was scored either as 1 = risk factor present, or 0 = risk factor absent.^30^ Scores for all features were summed to create a final MetS score. Participants who had central obesity and a MetS score ≥2 were categorized as MetS cases.

### Assessment of PCOS features

PCOS features included biomarkers, symptoms, and behaviors associated with PCOS such as total testosterone (TT) concentrations, sex-hormone binding globulin (SHBG) concentrations, absence of menstruation (amenorrhea), and OCP use.^9,31,32^ While amenorrhea and OCP were self-reported by participants, TT and SHBG concentrations were assessed using blood draws according to NHANES protocol.^33^ The precision and accuracy for measuring SHBG and TT were found to be very high, particularly because NHANES implements well-standardized quality assurance and quality control protocols for ensuring the high-standard criteria for accuracy and precision. The detail of the sample preparation, data processing, method performance, quality assessment, and result calculation is given for TT.^33^ Briefly, testosterone levels were assessed using high-performance liquid chromatography coupled with tandem mass spectrometry.^33^ In this analysis, TT and SHBG concentrations were log-transformed to account for their skewed distributions.

### Statistical analyses

For this analysis, TT and SHBG values were log-transformed to account for their skewed distribution. Participant demographics were reported as either mean (standard error, SE) for continuous measures or frequency and weighted percentage for categorical characteristics. Bivariate associations between PCOS features and MetS with its components were assessed using weighted univariate logistic regression models with a type I error of 5%. Age-adjusted associations between PCOS features and MetS were determined using multivariable logistic regression models. Adjusted odds ratios (aOR), 95% confidence intervals (CI), and p-values were reported. A stratified analysis was conducted to examine race/ethnic group differences in the associations between PCOS features and MetS and its components. All analyses were performed using SAS version 9.4 (SAS Institute, Inc, Cary, NC, USA).

## Results

The final analytical sample consisted of 2,172 female participants with a mean age of 30.3 years (SE = 0.3). Approximately 59% of participants identified as NHW, 18.7% as Hispanic/Latina, 12.4% as Non-Hispanic Black (NHB), 6.2% as Non-Hispanic Asian, and 3.7% as other or multiple races (Table 1). The prevalence of MetS in this sample was 14.5%, and 52.3% had central obesity, 8.4% elevated blood pressure, 20.1% elevated fasting glucose, 29.9% low HDL cholesterol, and 10.6% elevated triglyceride. Mean TT and SHBG concentrations for the sample were 27.0 ng/dL and 77.4 nmol/L, respectively. Approximately 12.4% of participants reported experiencing amenorrhea, and 65.2% used OCP. In general, participants with MetS were older, had a higher BMI, lower mean TT and SHBG concentrations, and a higher prevalence of amenorrhea and OCP usage compared to those without MetS. Overall, participant age, BMI, race/ethnicity, TT, SHBG, amenorrhea, and OCP use were significantly associated with MetS (p < 0.01).

**Table 1.** Distribution of Participant Demographics and PCOS Features by Metabolic Syndrome Status (*N* = 2,172)

		Metabolic syndrome status		
	Overall (*n* = 2,172)	No (*n* = 1,878)	Yes (*n* = 294)	*p*-Value
Age (in years)^a^	30.3 (0.3)	29.2 (0.4)	36.8 (0.6)	<0.01^*^
Body mass index (kg/m^3^)^a^	27.7 (0.2)	26.4 (0.2)	35.9 (0.4)	<0.01^*^
Race and Ethnicity^b^				<0.01^*^
Hispanic/Latina	593 (18.7)	494 (17.9)	99 (23.2)	
Non-Hispanic White	713 (59.0)	602 (58.9)	111 (59.7)	
Non-Hispanic Black	488 (12.4)	432 (12.7)	56 (11.1)	
Asian	294 (6.2)	274 (6.7)	20 (3.1)	
Other/multi-race	84 (3.7)	76 (3.8)	8 (2.9)	
PCOS Features				
Total testosterone (TT)^a^	27.0 (0.6)	27.7 (0.7)	22.4 (0.7)	<0.01^*^
Sex hormone-binding globulin (SHGB)^a^	77.4 (2.4)	81.9 (2.7)	52.1 (3.1)	<0.01^*^
Amenorrhea (yes)^b,c^	185 (12.4)	142 (11.1)	43 (19.9)	<0.01^*^
Oral contraceptive use (Yes)^b,d^	1027 (65.2)	846 (63.3)	181 (75.6)	<0.01^*^
MetS Features				
Central obesity^b^	1063 (52.3)	769 (44.2)	294 (100)	<0.01^*^
Elevated blood pressure^b^	168 (8.4)	69 (3.6)	99 (36.4)	<0.01^*^
Elevated fasting glucose^b^	424 (20.1)	225 (11.9)	199 (68.1)	<0.01^*^
Low high-density lipoprotein (HDL) cholesterol^b^	646 (29.9)	390 (20.3)	256 (86.9)	<0.01^*^
Elevated triglyceride^b^	216 (10.6)	67 (3.7)	149 (51.7)	<0.01^*^

^a^ weighted mean (SE) reported.

^b^ frequency (weighted column %) reported.

^c^ *N* missing = 230.

^d^ *N* missing = 231.

^*^ Significant at α = 0.05.

LDL, low high-density lipoprotein; PCOS, polycystic ovarian syndrome; SHBG, sex hormone-binding globulin; TT, total testosterone.

Bivariate analysis of MetS and PCOS features showed significant associations between 3 PCOS features and central obesity (Table 2). The use of OCP [OR = 0.66; 95% CI: 0.54,0.80] was associated with lower odds of central obesity and every 1-unit increase in log-transformed SHBG (LSHBG) concentrations [β = −0.96; OR = 0.32; 95% CI: 0.24,0.42] was significantly associated with a lower likelihood of central obesity. However, those with amenorrhea were over two times more likely to have central obesity [OR = 2.19; 95% CI: 1.46, 3.30]. Similarly, the use of OCP and an increased concentration of log-transformed TT (LTT) and LSHBG concentrations were associated with lower odds of having most MetS components (Table 2). Although statistically insignificant, the exception to this trend was the 6% increase in the odds of low HDL among women who used OCP versus those who did not [OR = 1.06; 95% CI: 0.83,1.35]. Furthermore, experiencing amenorrhea significantly increased the odds of elevated blood pressure [OR = 2.05; 95% CI: 1.21,3.47], elevated fasting glucose [OR = 1.72; 95% CI: 1.11,2.66], low HDL [OR = 1.77; 95% CI: 1.27,2.47], and elevated triglyceride [OR = 1.80; 95%CI: 1.01,3.18].

**Table 2.** Crude and Adjusted Bivariate Associations of Polycystic Ovarian Syndrome Features and Components of the Metabolic Syndrome

	TT^a^	SHBG^a^	Amenorrhea	OCP use
Obesity				
Unadjusted	0.83 (0.67, 1.03)	0.32 (0.24, 0.42)*	2.19 (1.46, 3.30)*	0.66 (0.54, 0.80)*
Adjusted	1.07 (0.84, 1.36)	0.24 (0.18, 0.33)*	1.27 (0.84, 1.92)	1.17 (0.95, 1.42)
Elevated blood pressure				
Unadjusted	0.60 (0.41, 0.89)*	0.71 (0.49, 1.02)	2.05 (1.21, 3.47)*	0.38 (0.25, 0.57)*
Adjusted	0.98 (0.66, 1.47)	0.56 (0.35, 0.90)*	0.92 (0.53, 1.60)	0.74 (0.47, 1.18)
Elevated fasting glucose				
Unadjusted	0.72 (0.53, 0.97)*	0.51 (0.36, 0.73)*	1.72 (1.11, 2.66)*	0.83 (0.61, 1.13)
Adjusted	0.91 (0.66, 1.25)	0.44 (0.30, 0.64)*	1.06 (0.70, 1.60)	1.39 (1.03, 1.88)*
Low HDL cholesterol				
Unadjusted	0.58 (0.46, 0.73)*	0.29 (0.23, 0.36)*	1.77 (1.27, 2.47)*	1.06 (0.83, 1.35)
Adjusted	0.60 (0.47, 0.76)*	0.28 (0.22, 0.35)*	1.65 (1.18, 2.32)*	1.22 (0.94, 1.60)
Elevated triglyceride				
Unadjusted	0.48 (0.35, 0.66)*	0.66 (0.41, 1.07)	1.80 (1.01, 3.18)*	0.55 (0.39, 0.77)*
Adjusted	0.58 (0.41, 0.81)*	0.59 (0.35, 0.99)*	1.17 (0.65, 2.09)	0.81 (0.58, 1.14)

^a^ log transformed to counter skewed distribution.

^*^ *p* < 0.05, statistically significant at type I error of 5%.

HDL, high-density lipoprotein; OCP, oral contraceptive pills.

After adjusting for age, the odds of central obesity [β = −1.19; aOR = 0.24; 95% CI: 0.18,0.33], elevated blood pressure [aOR = 0.56; 95% CI: 0.35,0.90], and elevated fasting glucose [β = −0.82; aOR = 0.44; 95% CI: 0.30,0.64] significantly reduced with an increase in LSHBG concentrations (Table 2). Every 1-unit increase in LTT and LSHBG concentrations was significantly associated with lower odds of low HDL and elevated triglyceride, while amenorrhea increased the odds of low HDL by 65% [aOR = 1.65; 95% CI: 1.18,2.32] (Table 2). These findings aligned with the adjusted analysis of the overall association of MetS with PCOS features; increases in LTT and LSHBG concentrations were significantly associated with lower odds of MetS (Table 3); there was a 42% reduction [β = −0.54; aOR = 0.58; 95% CI: 0.43,0.80] in the odds of MetS with every 1-unit increase in LTT and a 77% [β = −1.46; aOR = 0.23; 95% CI: 0.16,0.34] reduction in the odds of MetS with every 1-unit increase in LSHBG concentrations (Table 3).

**Table 3.** Age-Adjusted Associations Between Polycystic Ovarian Syndrome Features and Metabolic Syndrome

	Odds ratio [95% CI]	*p*-Value
Log (total testosterone)	0.58 (0.43, 0.80)	<0.01*
Log (SHBG)	0.23 (0.16, 0.34)	<0.01*
Amenorrhea (yes vs. no)	1.10 (0.73, 1.66)	0.64
Oral contraceptive (yes vs. no)	1.03 (0.81, 1.33)	0.79

^*^ *p* < 0.05; significant at type I error of 5%.

CI, confidence intervals.

In the context of race and ethnicity, results showed that increased concentrations of LSHBG were largely protective against MetS for most race/ethnicity groups except among Asians [β = −0.58; aOR = 0.56; 95% CI: 0.10,3.26]. Additionally, LTT was protective against MetS only among NHWs [β = −0.75; aOR = 0.47; 95% CI: 0.30,0.75]. Conversely, amenorrhea was also significantly associated with MetS, but only among participants who were of other/multiple races.

When analyzing associations with individual MetS components, increased LSHBG concentrations were associated with lower odds of central obesity and low HDL across all race/ethnicity groups. Increased LSHBG concentration was also associated with lower odds of elevated blood pressure and elevated fasting glucose across various races/ethnicities (Table 4). The most significant protective effect of increased LSHBG concentrations against central obesity was observed among women of other/multi-race [aOR = 0.01; 95% CI: 0.01,0.14], followed by NHW, Hispanic/Latinas, NHB, and Asians. Additionally, low LSHBG concentrations were protective against elevated blood pressure by 57% in NHW [aOR = 0.43; 95% CI: 0.20,0.90] and 74% other/multi-race [aOR = 0.26; 95% CI: 0.12,0.58] (Table 4). Increased LTT concentrations were associated with lower odds of central obesity among other/multiple races [aOR = 0.53; 95% CI: 0.32,0.87] but higher odds among Hispanics/Latinas [aOR = 1.39; 95% CI: 1.03,1.86]. Additionally, increased LTT concentrations were associated with a lower likelihood of having elevated fasting glucose in Hispanics/Latinas [aOR = 0.68; 95% CI: 0.48,0.94] and other/multi-race women [aOR = 0.48; 95% CI: 0.29,0.82], low HDL in all race/ethnicity groups except NHB and Asians, and reduced the odds of elevated triglycerides by among NHW [aOR = 0.50; 95% CI: 0.28,0.91] and other/multi-race individuals [aOR = 0.61; 95% CI: 0.39,0.97] (Table 4).

**Table 4.** Associations Between Polycystic Ovarian Syndrome Features and Metabolic Syndrome Components Stratified by Race/Ethnicity

	TT^a^	SHBG^a^	Amenorrhea	OCP Use
Metabolic Syndrome				
Hispanic/Latina	0.68 (0.44, 1.05)	0.32 (0.20, 0.51)^*^	1.28 (0.62, 2.65)	0.97 (0.63, 1.52)
Non-Hispanic White	0.47 (0.30, 0.75)^*^	0.21 (0.11, 0.39)^*^	1.13 (0.65, 1.96)	1.07 (0.67, 1.73)
Non-Hispanic Black	1.01 (0.58, 1.75)	0.30 (0.12, 0.73)^*^	0.51 (0.21, 1.23)	0.60 (0.28, 1.32)
Asian	0.44 (0.11, 1.76)	0.56 (0.10, 3.26)	0.84 (0.10, 7.48)	0.80 (0.22, 2.84)
Other/multi-race	0.65 (0.33, 1.27)	0.03 (0.01, 0.18)^*^	2.79 (1.04, 7.52)^*^	0.54 (0.10, 2.81)
Obesity				
Hispanic/Latina	1.39 (1.03, 1.86)^*^	0.30 (0.17, 0.55)^*^	1.47 (0.78, 2.75)	1.34 (0.88, 2.04)
Non-Hispanic White	1.01 (0.70, 1.44)	0.19 (0.12, 0.31)^*^	1.26 (0.75, 2.13)	1.38 (0.99, 1.92)
Non-Hispanic Black	1.25 (0.81, 1.93)	0.34 (0.20, 0.60)^*^	0.58 (0.24, 1.42)	0.65 (0.37, 1.14)
Asian	0.76 (0.39, 1.49)	0.44 (0.21, 0.92)^*^	1.14 (0.33, 3.94)	0.79 (0.39, 1.60)
Other/multi-race	0.53 (0.32, 0.87)^*^	0.01 (0.01, 0.14)^*^	9.50 (3.08, 29.37)^*^	3.11 (1.54, 6.28)^*^
Elevated Blood Pressure				
Hispanic/Latina	0.80 (0.39, 1.67)	0.71 (0.41, 1.22)	1.77 (0.63, 4.96)	1.29 (0.60, 2.78)
Non-Hispanic White	0.99 (0.53, 1.87)	0.43 (0.20, 0.90)^*^	0.79 (0.34, 1.85)	0.50 (0.19, 1.29)
Non-Hispanic Black	0.96 (0.52, 1.76)	0.97 (0.55, 1.72)	1.00 (0.41, 2.46)	0.65 (0.29, 1.43)
Asian	0.49 (0.09, 2.85)	0.60 (0.20, 1.80)	3.34 (0.42, 26.71)	0.58 (0.16, 2.03)
Other/multi-race	2.20 (0.12, 39.16)	0.26 (0.12, 0.58)^*^	^b^	0.82 (0.18, 3.80)
Elevated Fasting Glucose				
Hispanic/Latina	0.68 (0.48, 0.94)^*^	0.36 (0.22, 0.57)^*^	1.00 (0.41, 2.41)	1.88 (1.17, 3.01)^*^
Non-Hispanic White	1.07 (0.67, 1.71)	0.47 (0.29, 0.76)^*^	0.99 (0.58, 1.69)	1.46 (0.85, 2.52)
Non-Hispanic Black	0.96 (0.53, 1.74)	0.38 (0.17, 0.83)^*^	1.75 (0.77, 3.98)	0.70 (0.36, 1.39)
Asian	0.64 (0.33, 1.22)	0.63 (0.36, 1.10)	0.35 (0..04, 2.77)	1.14 (0.54, 2.45)
Other/multi-race	0.48 (0.29, 0.82)^*^	0.88 (0.54, 1.42)	3.09 (1.26, 7.58)^*^	0.74 (0.26, 2.12)
Low HDL Cholesterol				
Hispanic/Latina	0.59 (0.42, 0.83)^*^	0.33 (0.23, 0.47)^*^	1.75 (0.84, 3.65)	0.95 (0.65, 1.38)
Non-Hispanic White	0.56 (0.39, 0.83)^*^	0.26 (0.18, 0.37)^*^	1.80 (1.18, 2.75)^*^	1.58 (1.04, 2.39)^*^
Non-Hispanic Black	0.82 (0.57, 1.18)	0.44 (0.28, 0.69)^*^	0.60 (0.27, 1.35)	0.77 (0.44, 1.32)
Asian	0.56 (0.26, 1.21)	0.34 (0.20, 0.58)^*^	0.85 (0.16, 4.41)	1.51 (0.84, 2.69)
Other/multi-race	0.47 (0.30, 0.73)^*^	0.03 (0.01, 0.15)^*^	4.38 (2.12, 9.05)^*^	0.80 (0.46, 1.40)
Triglyceride				
Hispanic/Latina	0.70 (0.47, 1.05)	0.57 (0.26, 1.28)	2.14 (0.93, 4.91)	0.67 (0.39, 1.14)
Non-Hispanic White	0.50 (0.28, 0.91)^*^	0.71 (0.35, 1.41)	1.06 (0.50, 2.24)	0.86 (0.53, 1.40)
None-Hispanic Black	0.64 (0.26, 1.57)	0.27 (0.03, 2.71)	^b^	0.61 (0.19, 1.97)
Asian	1.83 (0.79, 4.26)	0.68 (0.24, 1.95)	^b^	0.67 (0.28, 1.61)
Other/multi-race	0.61 (0.39, 0.97)^*^	0.33 (0.03, 3.44)	4.11 (0.88, 19.17)	0.34 (0.22, 0.54)^*^

^a^ log transformed to counter skewed distribution.

^b^ ORs could not be generated due to small sample size.

^*^ *p* < 0.05; significant at type I error of 5%.

Furthermore, experiencing amenorrhea significantly increased the odds of central obesity among other/multiple races [aOR = 9.50; 95% CI: 3.08,29.37], and elevated fasting glucose and low HDL in certain race/ethnicity groups; NHW had 1.80 times higher odds of low HDL [aOR = 1.80; 95% CI:1.18,2.75], and other/multi-race had 3.09 times higher odds of elevated fasting glucose [aOR = 3.09; 95% CI: 1.26,7.58] and 4.38 times higher odds of low HDL [aOR = 4.38; 95% CI:2.12,9.05], compared to those without amenorrhea (Table 4). OCP use had mixed effects on MetS components by race/ethnicity; we observed a significant increase in the odds of obesity [aOR = 3.11; 95% CI: 1.54,6.28] among other/multi-race women, elevated fasting glucose among Hispanic/Latina women [aOR = 1.88; 1.17,3.01], and low HDL among NHW women [aOR = 1.58; 95% CI: 1.04,2.39] who used OCP versus those who did not. Conversely, OCP use decreased the odds of elevated triglyceride by 66% in other/multi-race women [aOR = 0.34; 95% CI: 0.22, 0.54] (Table 4).

## Discussion

This study highlights the relationship between MetS, its components, and important biomarkers and clinical indicators of PCOS including TT, SHBG, amenorrhea, and OCP use. The findings emphasize the protective effect of increased LTT and LSHBG concentrations on the odds of MetS and presenting with specific MetS components while providing detailed insights into the magnitude and directionality of menstrual irregularities and OCP use on MetS. Additionally, this study is one of the first to look at the variations in the relationships between PCOS features and MetS and its components by race and ethnicity, addressing an important yet understudied aspect of racial disparities in women’s reproductive and cardiometabolic health.

Although it is largely known as a reproductive disorder, PCOS is also associated with endocrine functionality, hormonal pathways, and cardiovascular-related mortality.^34^ Previous research has consistently shown that women with PCOS are at a greater risk of developing MetS due to shared pathophysiological mechanisms, such as insulin resistance and hyperandrogenism.^35,36^ According to Armanini et.al, the hyperactivity of the hypothalamic-pituitary-gonadal axis results in excessive production of ovarian androgens by theca cells *via* the upregulation of CYP17A1 enzyme activity.^37^

Additionally, a study by Moghetti et.al suggests that hyperandrogenism promotes the accumulation of subcutaneous fat and insulin resistance by suppressing fat breakdown (lipolysis) and promoting the lipogenesis of adipocytes.^38^ Consequently, long-term accumulation of subcutaneous fat promotes insulin secretion and represses its clearance, inevitably leading to insulin resistance and the development of MetS.^39^ Additionally, hyperinsulinemia amplifies LH-induced androgen production in adipose cells and reduces the synthesis of SHBG in the liver,^40^ thus promoting higher plasma concentrations of androgens,^41^ another suspected cause of metabolic complication related to PCOS.^42^ Given the cyclic nature of these endocrine pathways, it is difficult to determine the start of the causal pathway. Based on current evidence, the relationship between MetS and PCOS seems bidirectional, given that MetS affects 33% of women with PCOS and that women with PCOS have an 11-fold higher risk of MetS.^36,43^

Regarding individual PCOS features and MetS components, studies have found low HDL to be the most prevalent MetS feature among women with PCOS, followed by central obesity, hypertriglyceridemia, elevated fasting glucose, and elevated blood pressure, respectively.^36^ Findings here were similar; central obesity was the most prevalent component, followed by low HDL, elevated fasting glucose, and elevated triglyceride. Additionally, our study found significant age-adjusted associations between LTT and MetS (*p* < 0.01), where participants with MetS had lower mean TT concentrations compared to those without MetS, and increased LTT levels were associated with lower odds of MetS. An NHANES study from 2011to 2016 had similar findings and authors noted an L-shaped, negative association between TT and MetS; an increase in TT reduced the odds of MetS by 30%–44%.^44^ This was also observed in another NHANES analysis of data from 2011to 2012,^45^ and confirmed by the findings of our study.

Another key finding of our analysis was the significant age-adjusted associations between MetS and LSHBG concentrations; on average, women with MetS had lower concentrations of SHBG compared to those without MetS, and increased LSHBG concentrations were associated with lower odds of MetS. These findings aligned with those of an Italian study, where women with decreased concentrations of SHBG had an increased risk of MetS (*p* < 0.0001).^46^ Longitudinal evidence also suggests a 39% reduction in the risk of MetS with higher plasma concentrations of SHBG [RR = 0.61; 95% CI:0.51,0.73], with the protective effect being marginally weaker among post-menopausal women [RR = 0.65; 95% CI: 0.51,0.81].^47^ However, there were other studies that found higher concentrations of SHBG to be associated with a higher risk of MetS (*p* < 0.05), but not with individual MetS components.^48^ However, they had a relatively small (*n* = 84) sample that also included non-female participants.

Apart from increased LTT and LSHBG concentrations, amenorrhea and OCP use are also well-recognized features of PCOS that are associated with adverse metabolic health outcomes in women.^1,8^ For instance, a cross-sectional analysis from the Korean NHANES study observed markedly higher odds of MetS [aOR = 2.52; 95% CI:1.54, 4.15], central obesity, elevated triglycerides, and low HDL-C among women who reported menstrual irregularity versus those who did not, after adjusting for age, alcohol consumption, smoking status, and physical activity.^49^ A smaller Brazilian study also noted significant increases in waist circumference (a measure of central obesity), 2-hour oral glucose tolerance test glucose levels, HOMA-IR, and triglycerides, and a decrease in serum concentrations of HDL-C among adolescents with irregular menstrual cycles.^50^ These observations concur with findings from our study, given that amenorrhea was more prevalent in participants who had MetS versus those who did not, and significantly increased the odds of having all MetS components.

Regarding OCP use, to our knowledge, only one major cross-sectional study conducted in Iran has examined the effects of OCP use on MetS and its components. The findings from this study showed 23% higher odds of MetS among women using OCP versus those who were not.^51^ Interestingly, the authors also noted an increase in the odds of MetS from 15% to 75% among OCP users with an increase in the number of MetS features present.^51^ Our analysis also showed an increase in the odds of Mets by 3% with the use of OCP, and significant, unadjusted protective effects of OCP use against central obesity, elevated blood pressure, and elevated triglycerides, but an adverse adjusted effect on fasting glucose. Therefore, prospective longitudinal studies with larger sample sizes and more robust data are needed to tease apart the underlying mechanism linking OCP use to MetS and to determine the true directionality of the causal pathway between OCP and MetS and its components.

Another important aspect to consider is the racial and ethnic disparities in PCOS features and MetS, which has been extensively discussed in previous literature. Studies have demonstrated that Hispanic/Latina women exhibit a higher prevalence of PCOS and are more susceptible to severe metabolic complications compared to NHW women.^1,52,53^ This is consistent with our findings, where Hispanic/Latina women with certain PCOS features had significantly higher odds of metabolic complications, including central obesity and elevated fasting glucose. Additionally, our study observed reduced odds of MetS among NHB women with amenorrhea, indicating potential protective PCOS-related factors unique to this group. These findings are consistent with existing research, in which controlled clinical trials found NHB women to have a significantly lower prevalence of MetS (24.5% vs. 42.2%), serum triglyceride levels (85.7 ± 37.3 vs. 130.2 ± 57.0 vs. 120.1 ± 60.5 mg/dL, *p* < 0.01), and hypertriglyceridemia (5.1% vs. 28.3% vs. 30.5%, *p* < 0.01) compared to both Hispanic/Latina and NHW women.^4^ They also noted less severe PCOS phenotypes in NHB women compared to Hispanic/Latina, in certain respects, NHW women.^4^

Collectively, current literature and the findings from this study emphasize the importance of considering metabolic and cardiovascular health in the context of PCOS care, while accounting for racial and ethnic variations in physiology and body composition. The public health and policy implications of these findings are crucial, advocating for tailored approaches to MetS screening especially for individuals who present with features of PCOS. Given the racial disparities in the effects of PCOS features on MetS, culturally competent strategies are needed to effectively educate individuals with PCOS on their risk of MetS or individual MetS components and prevent them from developing metabolic complications such as obesity, insulin resistance, and diabetes.

### Strengths and limitations

The advantages of this study lie in its use of a large, nationally representative dataset (NHANES), which allows for the generalization of findings to the broader US population. The inclusion of racially and ethnically diverse participants enhances the study’s relevance and applicability to various demographic groups. Furthermore, the study’s rigorous statistical methods, including the use of log-transformation PCOS biomarkers with skewed distribution and age-adjusted multivariable logistic regression models, strengthen the validity of the findings. These strengths highlight the importance of considering racial and ethnic disparities in health research and underscore the need for targeted interventions to address the unique needs of diverse populations.

Despite the insightful findings of this study, several limitations should be acknowledged. A primary limitation is the absence of a variable for clinically diagnosed PCOS in the NHANES dataset. This absence is likely a result of the broad and varied diagnostic criteria for PCOS and the absence of data for one or more criteria, which complicates the direct identification of individuals with the condition. Consequently, we relied on surrogate biomarkers of PCOS such as TT, SHBG concentrations, and amenorrhea, which may not capture the true prevalence of the condition. The use of surrogate markers has previously been shown to be less consistent in predicting the morbidity of a health condition, thus leaving some room for biased measures of association. Furthermore, while this study provides important insights into the relationship between MetS and PCOS, it does so in a sample of women who did not have type 2 diabetes. Women with type 2 diabetes were excluded from the sample because of their distinct metabolic profiles that could introduce heterogeneity into the analysis. Given that participants would have to meet the elevated glucose levels of MetS to be diagnosed with type 2 diabetes, including women with type 2 diabetes in the sample would have artificially inflated the study’s MetS estimates. Therefore, the findings from this study should be interpreted with caution and not generalized to women with type 2 diabetes. Additionally, the high prevalence of OCP use in our sample may have artificially increased plasma SHBG concentrations, indirectly influencing the association between SHBG and MetS. Future research may benefit from examining the association of SHBG and MetS in populations with lower or no OCP use, to determine the extent to which OCP may confound or modify the above association. The cross-sectional design of this study restricted our ability to establish a causal pathway between PCOS features and MetS and determine the directionality of causality. Furthermore, the directionality of the associations between PCOS features and MetS/MetS components could not be determined.

Future longitudinal studies are necessary to establish a causal pathway linking PCOS with cardiometabolic health outcomes like MetS. While cross-sectional data are useful for determining the presence of associations, they are limited in their insight on causality and temporality of exposure and outcome. A prospective design would be most appropriate to determine how the underlying endocrine functions associated with PCOS features contribute to the development of MetS and its components over time. This will facilitate the development of targeted interventions and preventative strategies, potentially improving the long-term health outcomes for women with PCOS.

## Conclusions

In conclusion, this analysis revealed significant associations between PCOS features and MetS among a racially and ethnically diverse population of reproductive-aged women in the United States. Notably, increased LTT and LSHBG concentrations were associated with lower odds of MetS and its features, highlighting their critical role in cardiometabolic health. Amenorrhea was found to significantly increase the risk of MetS and its components, underscoring the importance of menstrual regularity in cardiometabolic health management. Furthermore, racial and ethnic disparities were evident, indicating a need for tailored and culturally competent clinical and healthcare approaches.

## Data Availability

Data used in this analysis is available upon reasonable request addressed to the corresponding author.

## Abbreviations Used

BMI: Body mass index
CDC: Centers for Disease Control and Prevention
CI: Confidence interval
IRB: Institutional Review Board
LSHBG: Log-transformed Sex-Hormone Binding Globulin
LTT: Log-transformed Total Testosterone
MEC: Mobile Examination Center
MetS: Metabolic Syndrome
NHA: Non-Hispanic Asian
NHANES: National Health and Nutrition Examination Survey
NHB: Non-Hispanic Black
NHW: Non-Hispanic White
NIH: National Institutes of Health
OCP: Oral Contraceptive Pill
OR: Odds ratio
PCOS: Polycystic Ovary Syndrome
PSU: Primary sampling units
SAS: Statistical Analysis Software
SD: Standard deviation
SE: Standard error
SHBG: Sex hormone-binding globulin
TT: Total Testosterone
U.S.: United States
